# Construction of immunotherapy-related prognostic gene signature and small molecule drug prediction for cutaneous melanoma

**DOI:** 10.3389/fonc.2022.939385

**Published:** 2022-07-25

**Authors:** Jiahua Xing, Ziqi Jia, Yan Li, Yan Han

**Affiliations:** ^1^ Department of Plastic and Reconstructive Surgery, The First Medical Center, Chinese PLA General Hospital, Beijing, China; ^2^ School of Medicine, Nankai University, Tianjin, China; ^3^ Peking Union Medical College, Chinese Academy of Medical Sciences, Beijing, China

**Keywords:** cutaneous melanoma, tumor microenvironment, prognostic signature, immunotherapy, bioinformatics

## Abstract

**Background:**

Cutaneous melanoma (CM), a kind of skin cancer with a high rate of advanced mortality, exhibits a wide variety of driver and transmitter gene alterations in the immunological tumor microenvironment (TME) associated with tumor cell survival and proliferation.

**Methods:**

We analyzed the immunological infiltration of TME cells in normal and malignant tissues using 469 CM and 556 normal skin samples. We used a single sample gene set enrichment assay (ssGSEA) to quantify the relative abundance of 28 cells, then used the LASSO COX regression model to develop a riskScore prognostic model, followed by a small molecule drug screening and molecular docking validation, which was then validated using qRT-PCR and IHC.

**Results:**

We developed a prognosis model around seven essential protective genes for the first time, dramatically elevated in tumor tissues, as did immune cell infiltration. Multivariate Cox regression results indicated that riskScore is an independent and robust prognostic indicator, and its predictive value in immunotherapy was verified. Additionally, we identified Gabapentin as a possible small molecule therapeutic for CM.

**Conclusions:**

A riskScore model was developed in this work to analyze patient prognosis, TME cell infiltration features, and treatment responsiveness. The development of this model not only aids in predicting patient response to immunotherapy but also has significant implications for the development of novel immunotherapeutic agents and the promotion of tailored treatment regimens.

## Introduction

The incidence of cutaneous melanoma (CM), a malignant tumor of the skin and mucous membranes, has been increasing globally over the past few decades. There were more than 100,000 cases of CM recorded in the United States in 2021 (1). Although CM accounts for only 5% of all skin cancers, it is responsible for up to 80% of skin cancer mortality (2). The incidence of CM is rising by 3% to 7% annually, posing a serious threat to human life and health (3). Overall 10-year survival statistics for patients with early-stage CM (stages I and II) remain favorable, ranging between 75% and 94% (4). In general, early-stage CM has a high cure rate after complete resection. In contrast, the mortality rate of patients with advanced CM (stages III and IV) is up to 70%, and the 5-year survival rate is less than 16% (5). CM comprises a small proportion of all skin tumors; compared to other skin tumors. It has clinical features such as high malignancy, high recurrence rate, facile metastasis, high late mortality, and high therapeutic resistance (6–8). Despite increased clinical attention, CM’s clinical efficacy and patient prognosis have not met expectations due to its complicated genetic and molecular mechanisms (9).

With the advent of BRAF inhibitors, the therapy and management of CM changed dramatically; since then, an increasing number of immune checkpoint inhibitors (ICIs) have been employed in the treatment of CM (10, 11). In a minority of CM patients with lasting responses, immunotherapies such as ICIs (anti-PD-1/L1 antibodies and anti-CTLA-4 antibodies) have a favorable prognosis. However, most patients do not have a favorable prognosis from them. This difference in clinical response rates across tumors of the same and different types indicates that tumor tissue has innate and acquired immunological resistance to immune checkpoints (12–14). Many researchers currently believe that the tumor microenvironment (TME) is a network of tumor cells and stromal cells (fibroblasts, vascular cells, and inflammatory immune cells) that play an important role in immune evasion and immunotherapy resistance (15). In recent years, an increasing number of studies have focused on TME to construct prognostic models with excellent performance and clinical application by comprehensively portraying the immune infiltration landscape (16–19). Therefore, this study employed immune-related gene sets in combination with clinical data from multiple databases to construct a prediction model with TME immune cell infiltration features at its core. In addition, this study employed highly expressed tumor-protective genes as the core of the model for the first time, as well as small molecule therapeutic screening and molecular docking validation.

## Material and method

### Gene expression profiles of CM and normal skin tissue

We first downloaded the TCGA TARGET GTEx dataset from the UCSC database (https://xena.ucsc.edu/). We extracted the normal group in TCGA and the normal skin data in GTEx as the control group. Correspondingly, we used TCGA-SKCM as the tumor group. What’s more, we utilized GSE54467 as a validation group. Our study included 1025 CM expression profile cohorts, including the TCGA and GTEx. Using the R package TCGAbiolinks, we obtained the COUNT gene expression values from public genomic data (20). Our inclusion criteria were: (i) CM diagnosed by histopathological examination in accordance with the International Classification of Diseases, Oncology, Third Edition (ICD-O-3-8772/3,8773/3,8774/3) classification. (ii) Melanoma whose primary site is located in the skin (melanoma of the skin in the World Health Organization 2008 site code, the corresponding primary site code is C49.0-49.6). Exclusion criteria were: (i) Cases with incomplete clinical information, such as race, American Joint Committee on Cancer (AJCC) stage, TNM stage, site, tumor thickness, and whether ulceration was unknown were excluded. (ii) Patient reports were limited to autopsy and death certificates. (iii) The cause of death was not known. This study included a total of 469 CM samples and 556 normal samples. We utilized the R package Combat algorithm to correct for batch effects due to abiotic bias. In addition, we downloaded immune-related genes from the immport (https://www.immp ort.orgc) database for subsequent analysis.

### Screening of immune-related differentially expressed genes (DEGs)

To identify key molecules associated with patients’ prognosis and TME cellular immune infiltration characteristics, we identified differentially expressed genes (DEGs) in CM and normal skin tissue using the empirical Bayes method in the R language limma-voom package. We used |logFC|≥2 and a false discovery rate adjust *p*-value<0.01 as a cut-off criterion to screen significantly DEGs. In addition, we utilized the R package ClusterProfiler for gene ontology (GO) and Kyoto Encyclopedia of Genes and Genomes (KEGG) enrichment analysis to investigate further the potential biological processes related to immune-related DEGs (20).

### Identification of key molecules

First, we submitted all DEGs to the STRING database to generate network maps of their protein-to-protein interactions (PPI). Then, we identify significant sub-network modules from the PPI network using the MCODE plugin for Cytoscape. We established the cutoff criterion as follows: degree cutoff=10, node score cutoff=0.2, k-core=2, max.depth=100. We will choose genes with high connection and significant predictive value (*P*<0.01) among significant subnetwork modules as important molecules (21). Finally, we chose CD86, CXCL9, FCGR3A, GZMB, PRF1, STAT1, and TLR7 as our key molecules. We utilized TCGA mutation data to show key molecules’ mutation frequencies and mutation types in CM patients. A total of 467 CM patients with complete clinical annotation were available for CM survival analysis. We utilized the R package survminer to determine appropriate cut-off points, classified patients into high- and low-risk groups and compared the protein expression of key molecules in CM and normal skin tissue using the Human Protein Atlas (https://www.prote inatlas.org/).

### Extrapolation of TME infiltrating cells

We utilized single sample gene set enrichment analysis (ssGSEA) to estimate the infiltration abundance of each TME cell based on the Cibersort gene set (22). To control the bias induced by tumor purity, we employed the ESTIMATE algorithm to accommodate the enrichment scores for each TME cell subtype (23). We employed 28 human TME cell subtypes and expressed the abundance of each TME-infiltrating cell by the adjusted enrichment scores determined using ssGSEA.

### Construction of a key molecules-based prognostic model

We constructed riskScore models based on the involvement of these seven critical genes in the course of CM to analyze the relevance of these molecules in patient prognosis, TME immune cell infiltration, and immunotherapy responsiveness. We created prognostic models for fitting the overall survival (OS) of CM patients using least absolute shrinkage and selection operator (LASSO) Cox regression analysis. In order to construct the optimal prognostic model, we utilized the R language’s glmnet package to select and reduce the variables so that some of the regression coefficients were strictly equal to 0. In addition, we employ 10 cross-validations to establish the penalty parameter (λ) of the prognostic model and adhere to the minimum criterion (the value of λ corresponds to the lowest likelihood deviation) (24). The riskScore is defined as $riskScore=\sum_{i=1}^{n}Coefficient\times Expression$ . The coefficient is defined as the LASSO COX regression coefficient, and Expression is defined as the expression of key genes. Furthermore, we utilized GSE54467 as a validation group to verify the performance of our riskScore.

### Access to immunotherapy cohort and clinical information

We included the IMvigor210 immunotherapy group from previous studies with complete clinical and transcriptome data in our analysis (25). The IMvigor210 cohort focuses on the efficacy of an anti-PD-L1 antibody (pembrolizumab) in patients with advanced uroepithelial carcinoma. IMvigor210 cohort has been widely used in lung adenocarcinoma (26), colon cancer (27), breast cancer (28), hepatocellular carcinoma (29), and head and neck squamous cell carcinoma (30) as a high-quality and comprehensive immunotherapy cohort to evaluate the predictive effect of immunotherapy in different types of tumor prediction models. We downloaded the complete transcriptomic data and detailed clinical information from the relevant URL (http://research-pub.gene.com/IMvigor210CoreBiologies/). Afterward, using the R package DEseq2, we normalized the data and transformed the count values to TPM values.

### Chemotherapy drug sensitivity analysis, small molecule drug screening and molecular docking validation

First, we utilized the R package pRRophetic to examine the half-maximal inhibitory concentrations (IC50) of common chemotherapeutic agents and targeted medicines to quantify our riskScore model’s prediction power for CM treatment. Following that, we calculated medications with significantly negative correlations with seven highly elevated genes using the Cmap (Connectivity Map) database and then selected the top 10 drugs (31). Furthermore, we obtained the SDF 2D structure files of the 10 drug candidates from the Pubchem database, transformed the small molecules into 3D structure files using Autodock MGLTools, performed energy optimization, and then exported the files in PDBQT format. We downloaded seven highly up-regulated genes from the PDB database to determine crystal structures. However, CXCL9 and PRF1 were excluded from the subsequent molecular docking investigation because they lacked crystal structure information. The receptor crystal structures were processed in bulk using the prepare_recpetor4.py script in Autodock MGLTools and then docked to small molecules and receptor proteins using Autodock Vina (version 1.1.2). In addition, Pymol was employed to map the small molecule-protein binding and visualize molecular docking results (32). Finally, the ComplexHeatmap R package was employed to generate the docking scoring heat map.

### Validation of key molecules in cells and tissues

We utilized the A375 human melanoma cell line, SK-MEL-28 human melanoma cell line, human immortalized keratin-forming cell line (Hacat), and human melanocyte cell line (PIG1) to verify important genes in CM and normal skin tissues. All cells were grown in RPMI-1640 media supplemented with 10% fetal bovine serum in a 37°C, 5% CO2 atmosphere.

Then, we obtained 20 fresh frozen CM tumor tissue and normal skin tissue specimens for self-matching and separated them into the tumor and normal groups. The specimens and patients were in one-to-one correspondence. The Human Research Ethics Committee of the Chinese PLA General Hospital authorized all experimental components, and patients signed informed permission forms. We utilized qRT-PCR to detect the relative expression of seven key genes in fresh frozen specimens. Using Trizol reagent, we extracted total RNA from the four cell lines and tissues listed above. RNA concentration was determined using a NanoDrop spectrophotometer. We synthesized cDNA using PrimerScript 5×RT Master Mix (BioRad), and mRNA expression levels were quantified using a 2×SYBR Green PCR Kit based on fresh frozen specimens. GAPDH (glyceraldehyde-3-phosphate dehydrogenase) was utilized as an internal reference to normalize the mRNA expression levels of each gene. Utilizing the 2^-ΔΔCt^ approach, we quantified the real-time PCR analysis and determined the relative expression of essential genes individually in cells and human specimen tissues. Beijing Huada Corporation produced all primers. We presented the primer sequences and patient specimens’ information in **Supplemental Tables 1, 2**. In addition, we downloaded immunohistochemical (IHC) images of key genes’ CM and normal skin tissue from the Human Protein Atlas database. We selected a suitable field of view in each IHC image of normal skin tissues and CM for semi-quantitative analysis of protein expression levels using Image Pro Plus 6.0.

### Statistical analysis

We utilized the Wilcoxon test to analyze the differences between the two groups in this study. The Kruskal-Wallis test and one-way ANOVA were used to determine the significance of differences between three or more groups. Spearman’s analysis was used for correlation testing. Moreover, we utilized univariate Cox regression models to construct hazard ratios (HR) and 95 percent confidence intervals (95%CI). We also employed multivariate Cox model models to investigate the predictive potential of riskScore as an independent prognostic biomarker for assessing patient prognosis. All statistical *P* values in this investigation were two-tailed, and *P*<0.05 was considered statistically significant.

## Results

### Genomic mapping differences between normal and CM

**Figure 1:**
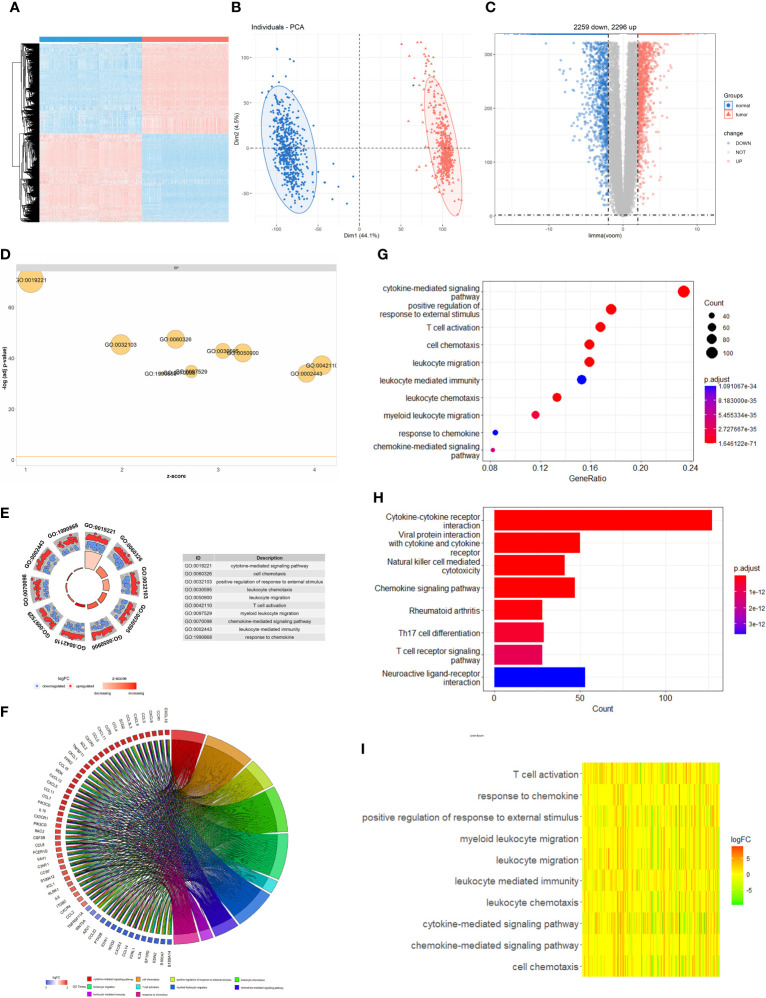
Difference of genomic landscape between normal and cutaneous melanoma. **(A)** Hierarchical clustering of differentially expressed genes between normal and cutaneous melanoma samples. Red represents up-regulated and blue represents down-regulated. **(B)** PCA visualization of differentially expressed genes. **(C)** Volcano plot of differentially expressed genes. **(D)** Biological processes enrichment of gene ontology functional enrichment. **(E)** Molecular function enrichment of gene ontology functional enrichment. **(F)** Chord plot of gene ontology functional enrichment. The left half-circle indicates that the genes are sorted by |logFC| and the right half-circle indicates that the gene ontology enrichment analysis term is sorted by strong and weak variation. Red represents up-regulation and blue represents down-regulation, and color shades represent fold change. **(G)** Cellular component enrichment of gene ontology functional enrichment. **(H)** KEGG pathway enrichment analyses for differentially expressed genes. All enriched pathways were significant and the color depth represented enriched adjusted *P* value. **(I)** Heatmap of differentially expressed genes between normal and cutaneous melanoma samples. Different colors represent different interaction strength relationships.

**Figure 2:**
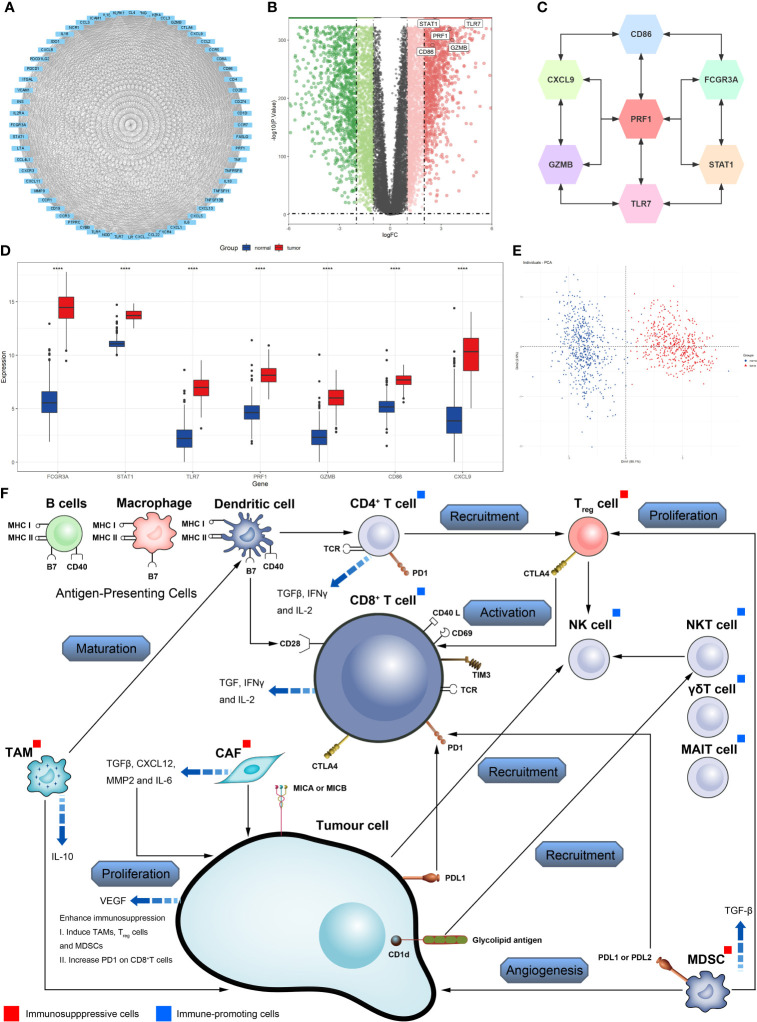
Identification of key molecules in cutaneous melanoma. **(A)** Construction of protein-protein interaction (PPI) network among differentially expressed genes. **(B)** Volcano plot constructed with the cut-off criterion *P*<0.05 and |logFC|≥1. **(C)** The relationship among the seven key molecules at the protein level, each gene is closely linked to each other at the protein level. **(D)** The seven key molecules expressed in the normal skin and cutaneous melanoma (****P <0.0001). **(E)** Principal component analysis for the key molecules revealed. This result shows that seven key molecules can distinguish very well between normal skin and cutaneous melanoma. **(F)** Effect of immune and stromal cells on cutaneous melanoma.

The flowchart of the study is depicted in **Supplementary Figure 1**. Using cluster analysis and principal component analysis (PCA), we first demonstrated the genetic differences between normal skin tissues and CM (**Figures 1A, B**). By comparing 469 CM and 556 normal skin tissues, we discovered that the expression of 4555 genes was significantly altered in tumor tissues against normal tissues (*P*<0.01, |logFC|≥2), with 2296 genes considerably up-regulated and 2259 genes significantly down-regulated (**Figure 1C**). The GO enrichment analysis shows that CM genetic variants are involved in TME immune components and matrix-related biological processes such as cytokine-mediated signaling, cell chemotaxis, and positive regulation of response to external stimulation (**Figures 1D–G**). According to the KEGG pathway, these genes also participate in immune-related signaling pathways such as cytokine-cytokine receptor interaction, viral protein interaction with the cytokine-cytokine receptor, viral protein interaction with cytokine and cytokine receptor, and natural killer cell-mediated cytotoxicity (**Figure 1H**).The heatmap results also imply that immunological and matrix-related pathways play a significant role in the CM genome (**Figure 1I**). The PPI network reveals a close interaction between CM-related genes at the protein level (**Figure 2A**). CD86, CXCL9, FCGR3A, GZMB, PRF1, STAT1, and TLR7 were identified as key molecules with strong connectivity in a sub-network module with solid predictive value. We also investigated the protein correlation between these key molecules (**Figures 2B, C**). We also discovered that the expression of key molecules in CM samples is substantially higher than in normal samples (**Figure 2D**). We performed dimensionality reduction using PCA to determine if these critical molecules can distinguish CM samples from normal samples. We discovered two completely disjoint populations, indicating that the expression patterns of critical molecules in normal and CM samples are distinct (**Figure 2E**). In addition, we demonstrated the interaction of numerous immune molecules in the CM TME, the signaling cascade and transmission, and the distinct regulation patterns between molecules (**Figure 2F**).

### Identify key molecules, mutation and survival analysis

**Figure 3:**
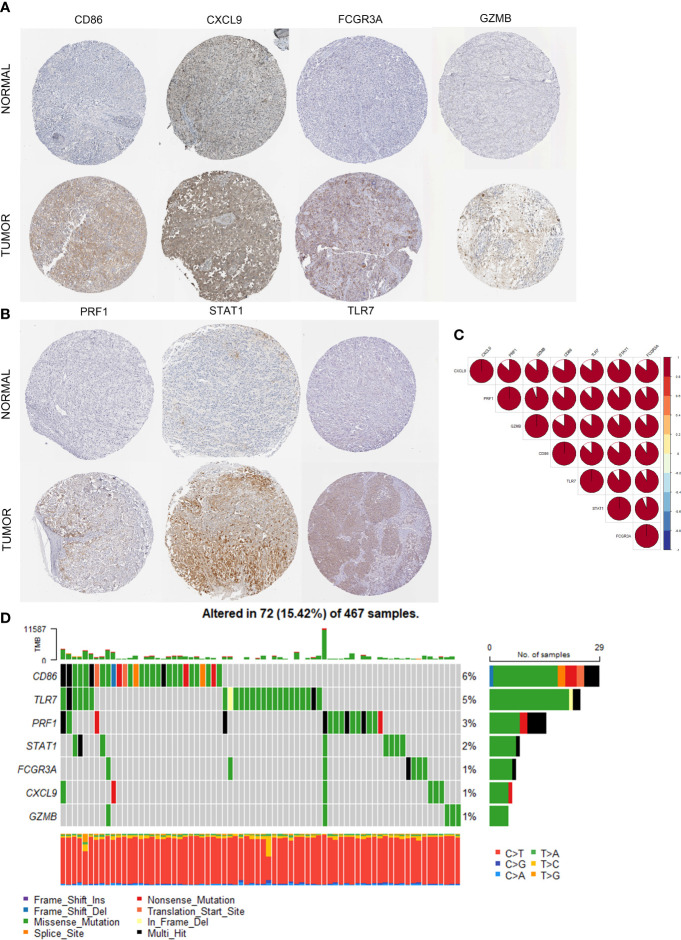
Multi-omics analysis of identified seven key molecules. **(A, B)** The immunohistochemical staining results revealed significant differences of key molecules (CD86, CXCL9, FCRG3A, GZMB, PRF1, STAT1, TLR7) at the protein expression between normal skin and cutaneous melanoma obtained at the Human Proteins Atlas. **(C)** The correlation between the seven key molecules using spearman analysis. The color area represents the magnitude of correlation intensity, red represents positive correlation and blue represents negative correlation. The key molecules in the figure show very good correlation with each other. **(D)** Mutation landscape of seven key molecules in 467 samples of TCGA cohort. Different color modules represent different molecular mutation frequencies.

**Figure 4:**
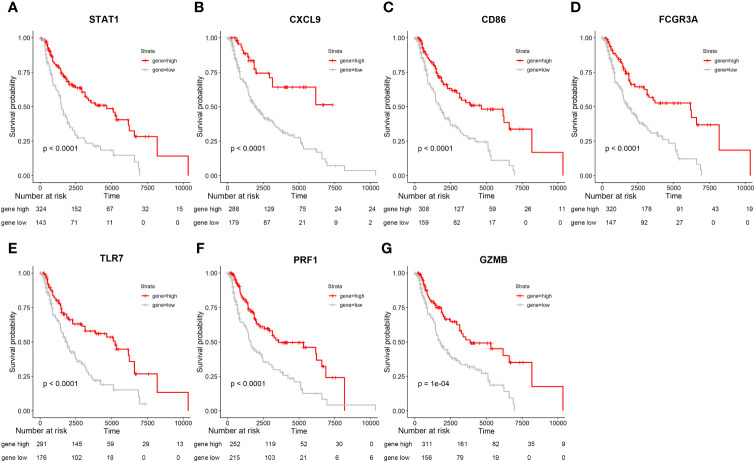
Seven Kaplan-Meier curves are based on samples from the GTEX and TCGA databases, extracting data from these seven genes and combining them with survival times. Specific information on the number of people is shown in the figure. **(A-G)** Survival analysis for seven key molecules. Seven key molecules which includes STAT1, CXCL9, CD86, FCGR3A, TLR7, PRF1, GZMB.

We obtained immunohistochemistry results from the HPA database for seven critical molecules and qualitatively identified protein-level expression variations between normal tissues and CM samples (**Figures 3A, B**). In order to validate the differential and substantial expression of key molecules in CM tissues, we examined the expression of these crucial molecules in cell lines and 20 pairs of tumors and surrounding normal tissues. Spearman correlation analysis identified a significant positive correlation between important molecules and a strong interaction between these molecules (**Figure 3C**). The mutations of CM’s essential molecules were then studied. Among the 467 patients with complete clinical annotation information, 72 patients (15.42%) had mutations in at least one gene locus. CD86 exhibited the highest frequency of mutations in CM samples, followed by TLR7, and all essential molecules were revealed to have gene mutations (**Figure 3D**). The expression and mutation of these essential molecules may play a crucial role in the growth and metastasis of CM, as deduced by our findings. In addition, we probed into the predictive value of key molecules based on an independent CM cohort from the TCGA database using survival analysis. The CM cohort in TCGA also demonstrated variations in the expression of essential molecules between normal and tumor samples. For survival analysis, 467 CM patients with complete clinical annotation were available. Patients with high expression of CD86, CXCL9, FCGR3A, GZMB, PRF1, STAT1, and TLR7 had a significant survival benefit over those with low expression (**Figures 4A–G**). Seven critical molecules displayed significantly greater expression levels in CM, and all seven key molecules as protective molecules significantly increased CM patient survival.

### Evaluation of immune cell infiltration characteristics of tumor microenvironment

**Figure 5:**
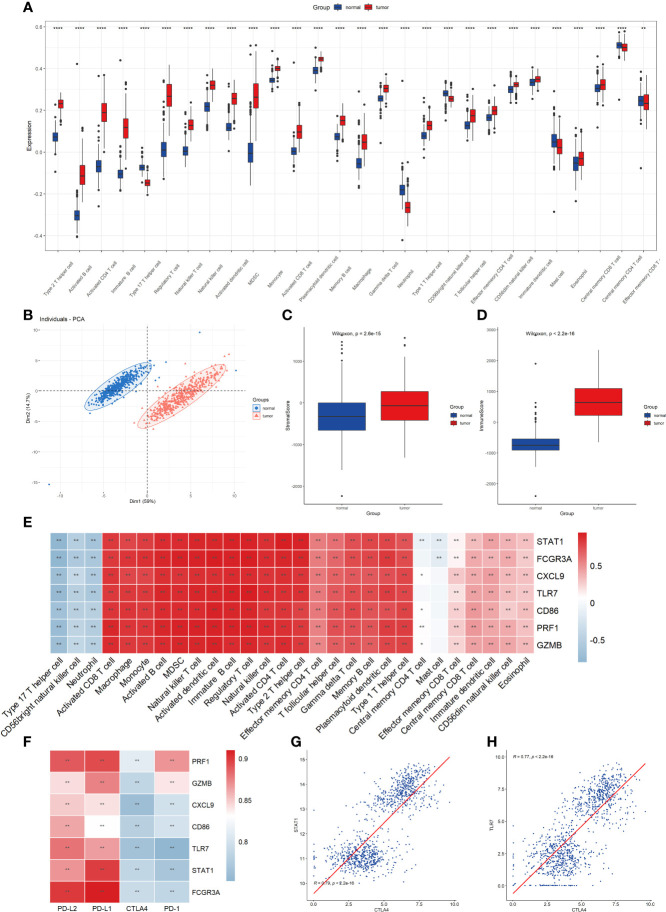
Evaluation of 28 TME immune cell infiltration characterization. **(A)** Differences in 28 TME infiltration cells between normal skin and cutaneous melanoma (**P <0.01, ****P <0.0001). The results showed that all immune cells were significantly different between the two types of samples. **(B)** Principal component analysis. The results demonstrated that the two separate taxa, suggesting there existed significantly differences in the landscape of 28 TME immune cell infiltration between normal skin and cutaneous melanoma. **(C)** Difference in StromalScore between normal and tumor tissues using ESTIMATE algorithm. **(D)** Difference in ImmuneScore between normal and tumor tissues using ESTIMATE algorithm. **(E)** The correlation between seven key molecule and each TME infiltration cell type. The results showed a strong correlation between them, red represents positive and blue represents negative. (*P<0.05, **P <0.01) **(F)** The correlation between the seven key molecules and four immune checkpoint molecules. The results demonstrated a strong correlation between them. (**P <0.01) **(G)** The correlation between STAT1 expression and CTLA4 expression. **(H)** The correlation between TLR7 expression and CTLA4 expression.

To further investigate the role of identified critical molecules in TME immune cell infiltration in CM patients, we analyzed the infiltration of 28 types of TME cells in normal and tumor tissues (**Figure 5A**). T helper cells (type 1 and 2), activated B cells, CD4^+^ T cells, CD8^+^ T cells, immature B cells, regulatory T cells, natural killer cell, activated dendritic cell, plasmacytoid dendritic cell, MDSC, monocyte, memory B cell, macrophage, gamma delta T cell, effector memory CD4^+^ T cells, CD56 dim natural killer cell, immature dendritic cell, eosinophil, and CD8^+^ T cells were all highly plentiful in tumor tissue. However, other cell subsets were notably abundant in normal tissue. Afterward, using the PCA algorithm, we compared the infiltration patterns of TME cells in normal and tumor tissues to determine if there were any differences. After dimensionality reduction, the results demonstrated the existence of two distinct populations of TME cells (**Figure 5B**). We then measured immunological and mesenchymal activity in the CM microenvironment using the ESTIMATE algorithm. It was revealed that immunological and mesenchymal activities were substantially greater in tumor tissues than in normal skin tissues (**Figures 5C, D**). To examine the link between critical molecules and immune cells in the TME, we correlated key molecules with cellular fractions in the TME. Spearman correlation analysis demonstrated that these molecules were highly positively associated with the majority of TME cellular fractions, except for T helper cells, CD56 bright natural killer cells, and neutrophil cell infiltration (**Figure 5E**). In addition, the expression of seven essential molecules demonstrated a significant positive association with PD-L2 and PD-L1 and a negative correlation with CTLA4 (**Figure 5F**). CTLA4 had the most significant connection with STAT1 and TLR7 (**Figures 5G, H**).

### Correlation model construction for prognosis and immunotherapy based on key molecules

**Figure 6:**
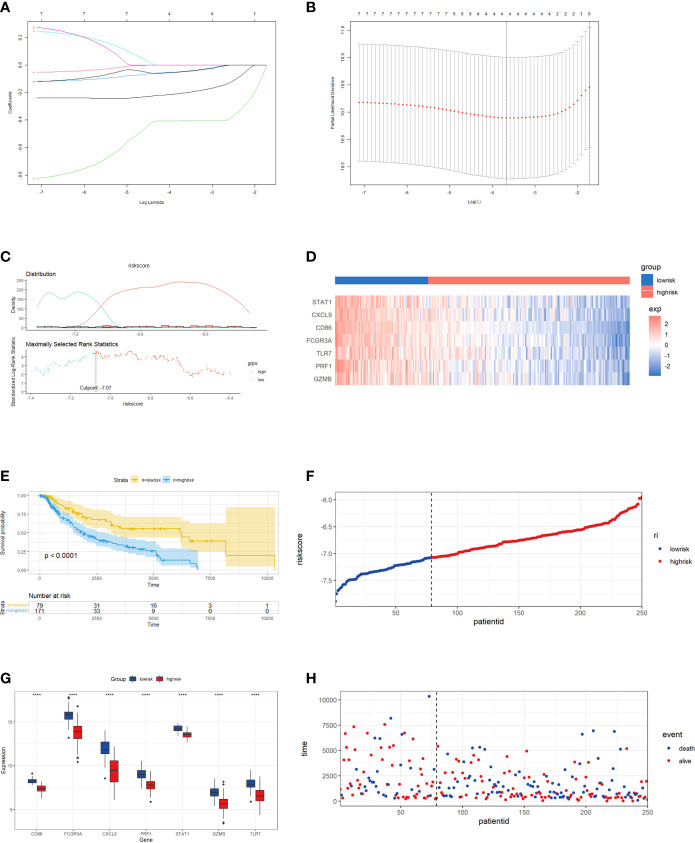
Construction of riskScore signature in cutaneous melanoma. **(A)** Least absolute shrinkage and selection operator (LASSO) coefficient profiles of the seven key molecules. Horizontal axis represents log of independent variable λ. Vertical axis represents coefficient of independent variable. **(B)** Tenfold cross-validation of tuning parameters in LASSO model. **(C)** The optimal cut-off point to dichotomize riskScore into low and high groups was determined by MaxStat R package. The optimal cutoff point was -7.07. **(D)** Survival analyses for low (79 samples) and high (171 samples) riskScore groups using Kaplan-Meier curves. **(E)** The seven key molecules expressed in the low and high risk groups (****P <0.0001). The results showed that a strongly significant difference was exhibited between the groups. **(F)** The median value and distribution of the risk score. **(G)** The distribution of overall survival (OS) status. **(H)** Hierarchical clustering of seven key genes between low and high risk groups. Red represents up-regulated and blue represents down-regulated.

**Figure 7:**
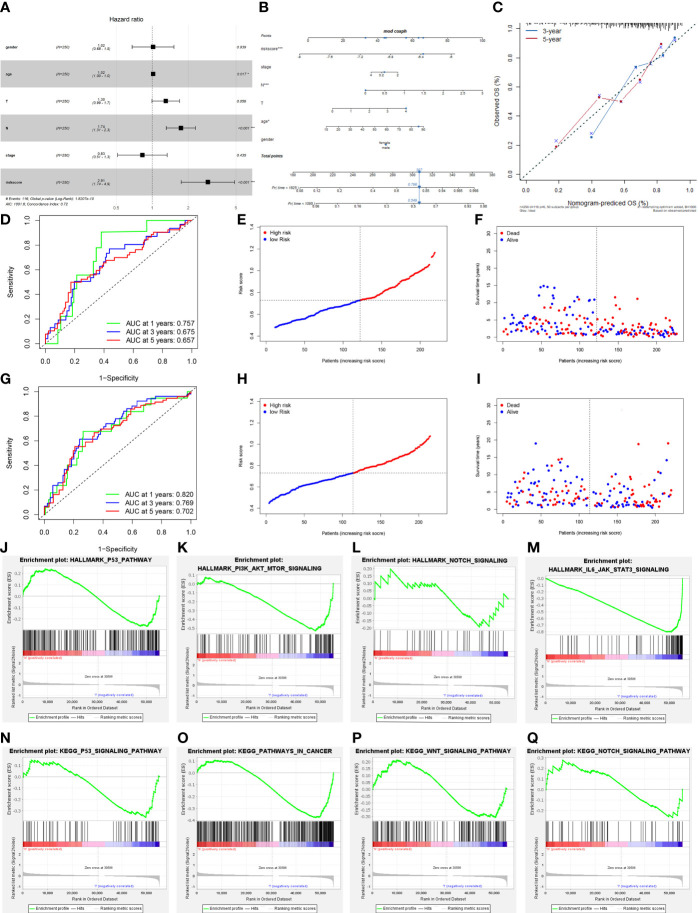
Prognostic value of the riskScore gene signature in cutaneous melanoma. **(A)** Forest plot. The results demonstrated that the riskScore and N were independent prognostic biomarkers using multivariate analyses. **(B)** The nomogram, including clinical features and the risk score, for predicting outcomes in patients. **(C)** The calibration curve analysis showed that the actual and the predicted 1-, 3-, 5-year survival times were consistent compared with the reference line (the 45-degree line). **(D)** The receiver operating characteristic curve (ROC) analysis of risk scores based on 1-, 3-, and 5-year OS in TCGA. **(E)** The median value and distribution of the risk score in TCGA. **(F)** The distribution of OS status in TCGA. **(G)** The ROC analysis of risk scores based on 1-, 3-, and 5-year OS in GSE54467. **(H)** The median value and distribution of the risk score in GSE54467. **(I)** The distribution of OS status in GSE54467. **(J–Q)** The GSEA enrichment reveal several significant signaling pathways. **(J)** HALLMARK P53 pathway. **(K)** HALLMARK PI3K AKT MTOR signaling pathway. **(L)** HALLMARK NOTCH signaling pathway. **(M)** HALLMARK IL-6 JAK STAT3 signaling pathway. **(N)** KEGG P53 pathway. **(O)** KEGG pathway in cancer. **(P)** KEGG WNT signaling pathway. **(Q)** KEGG NOTCH signaling pathway.

We incorporated patient prognostic information and TME immune cell infiltration status to build the riskScore model, and we integrated the role of these essential molecules using LASSO Cox regression. RiskScore was determined by the expression of the four most representative important molecules, according to the findings (**Figures 6A, B**). We classified the patients into high-risk and low-risk groups based on the critical value of -7.07 (**Figure 6C**) calculated using the MaxStat R package. We observed that the low-risk group had a considerable survival advantage over the high-risk group (**Figure 6D**). In addition, the expression of these essential molecules is significantly higher in low-risk tumors than in high-risk tumors, implying that these essential molecules have a protective role in the low-risk group, which is consistent with the findings of our previous study (**Figures 6E, F**). With rising risk, patient mortality might climb significantly (**Figures 6G, H**). Our examination of multivariate COX regression models incorporating basic clinical and pathological information about the patients demonstrated that riskScore could be an independent and robust predictive biomarker to evaluate CM patients (**Figure 7A**). Additionally, we developed a nomogram that combines the riskScore with independent clinical prognostic indicators to estimate the likelihood of patient mortality (**Figure 7B**). The calibration plots demonstrated that the generated nomogram had a superior prediction ability (**Figure 7C**). We displayed ROC curves based on TCGA data with AUC of 0.757, 0.675, and 0.657 for 1, 3, and 5 years. Then we use GSE54467 as a validation set to show the performance of our riskScore, with AUC of 0.820, 0.769, and 0.702 for 1, 3, and 5 years, indicating the riskScore’s predictive performance is acceptable and can provide a reference for clinical decision-making (**Figures 7D–I**).

**Figure 8:**
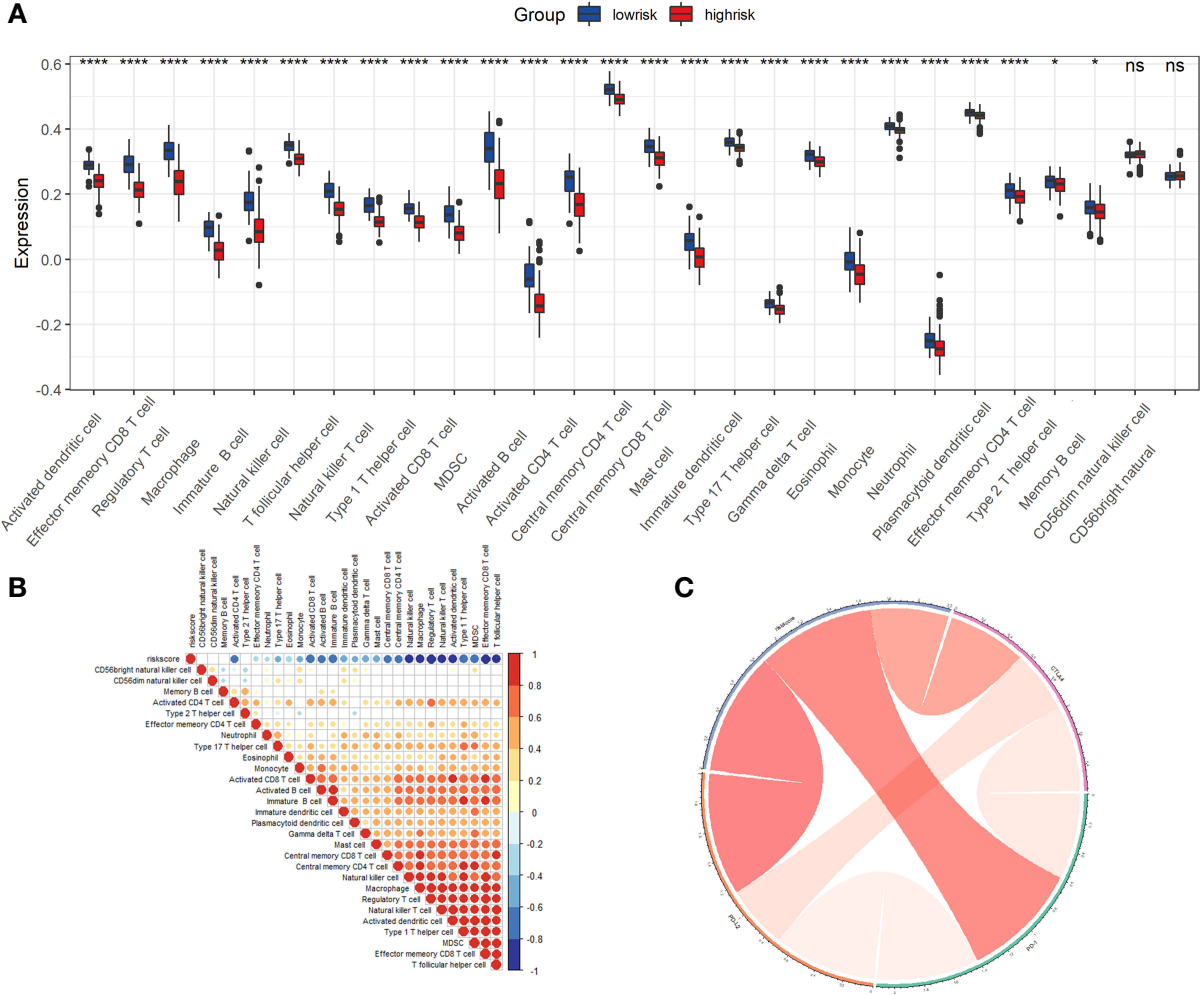
The role of riskScore signature in the TME cell infiltration and immunotherapeutic responses. **(A)** Differences in 28 TME infiltration cells between low and high risk groups ns, not significant, *P<0.05, ****P <0.0001. The results demonstrated that most TME cells (26 types) exist significant differences. **(B)** The correlation between riskScore signature and 28 TME cell infiltration. Color shades represent the strength of the association, blue represents negative correlation and red represents positive correlation. **(C)** The correlation between riskScore signature and immune checkpoint molecules. Blue represents negative correlation and red represents positive correlation. ns, not significant.

We utilized gene set enrichment analysis (GSEA) to investigate the activated biological pathways in the low-risk and high-risk groups. Compared to the low-risk group, cancer-related pathways such as P53, PI3K-AKT-mTOR, NOTCH, and WNT were considerably activated in the high-risk group (**Figures 7J–Q**). Then we analyzed the difference in TME cell infiltration between the low-risk and high-risk groups, and we discovered that all immune infiltrating cells, except for CD56 dim natural killer cells and CD56 bright natural killer cells, were significantly higher in the low-risk group than in the high-risk group (**Figure 8A**). We observed that riskScore values were strongly and positively related to the majority of TME cell infiltrating rates using correlation analysis (**Figure 8B**). We also discovered a significant and positive correlation between riskScore values and the expression of immune checkpoint molecules, indicating the potential predictive role of riskScore in predicting clinical response to immunotherapy and providing a foundation for developing novel immunotherapies (**Figure 8C**). As immune checkpoint blockade (ICB) has made advances in the treatment of CM over the past few years, we verified riskScore’s ability to predict the clinical response of patients to ICB therapy. Low-risk patients in the IMvigor210 cohort who received anti-PD-L1 treatment experienced significant clinical benefits and prolonged survival (**Supplementary Figure 2A**). The patients with complete remission (CR) or stable disease (SD) had a lower risk (**Supplementary Figure 2B**). Moreover, we noticed that low-risk individuals responded significantly better to PD-L1 blocking therapy than high-risk patients (**Supplementary Figure 2C**).

### Chemotherapy drug sensitivity analysis, small molecule drug screening and molecular docking validation

**Figure 9:**
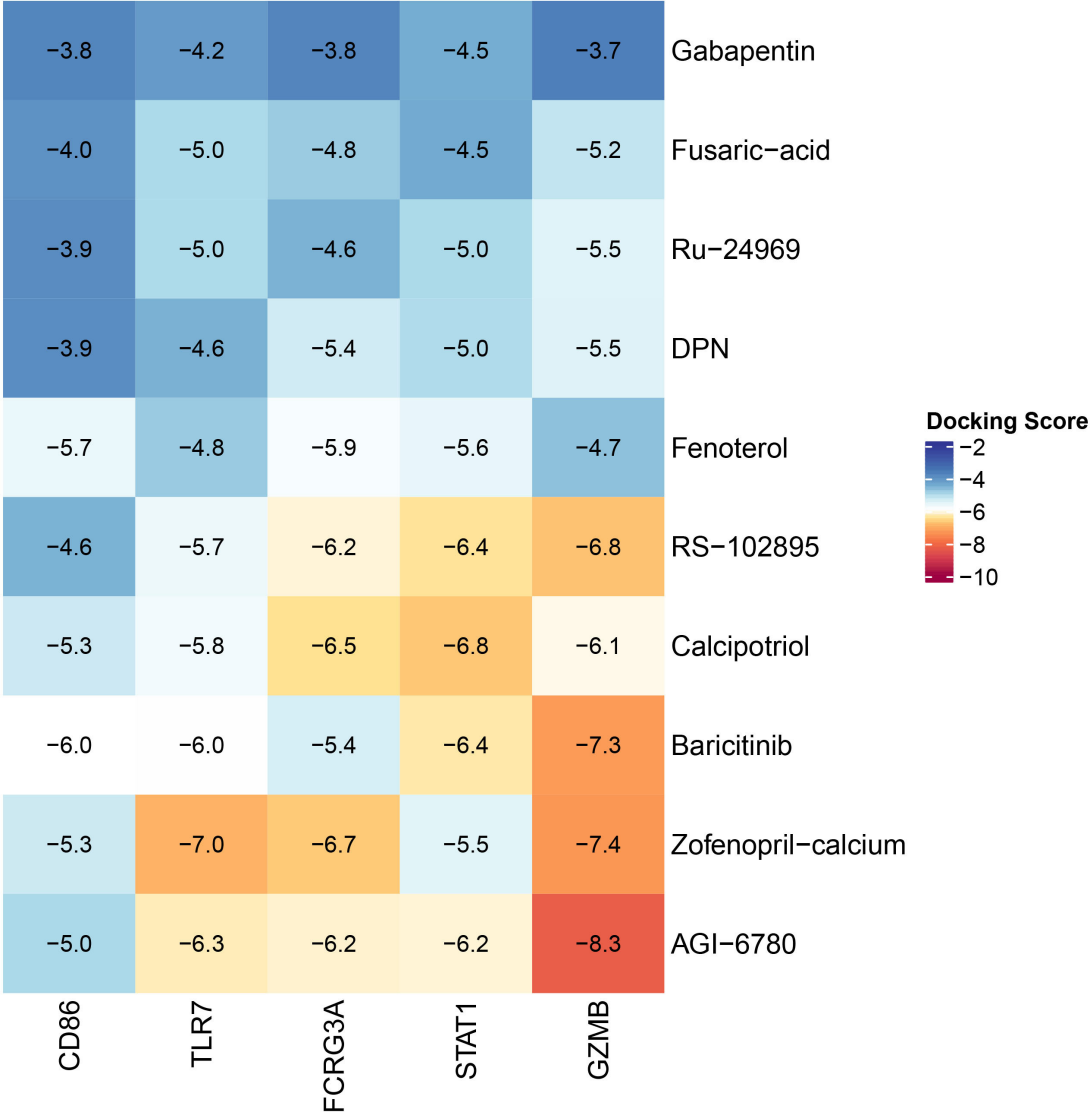
Heat map of the lowest binding energy for molecular docking.

We analyzed 20 common chemotherapeutic and targeted medications and discovered significant differences in IC50 values between the high-risk and low-risk groups (**Supplementary Figure 3**). The results indicate that our riskScore signature can uncover prospective biomarkers of chemotherapy and targeted medication sensitivity. Then, we calculated the connection between medication-treated expression profiles and highly up-regulated expression profiles of seven key genes using the Cmap database. We then identified the top ten pharmaceuticals with negative correlations as potential treatment candidates (**Table 1**). **Supplementary Figures 4A–J** shows the chemical structures of these ten compounds. AGI-6780 and Zofenopril-calcium bind well to GZMB, indicating that these two small compounds can be employed as possible target medicines to target GZMB. In addition, we utilized Pymol to generate a heatmap of CD86, FCGR3A, STAT1, TLR7, and GZMB protein binding to the most strongly bound small molecules or the top two most strongly bound small molecules (**Figure 9**). The results demonstrated that the small molecules of CD86 bound to Baricitinib formed hydrogen bonds with THR-69, SER-67, and GLN-16. The binding of FCGR3A to Zofenopril-calcium formed hydrogen bonds with HIS-111 and ARG-109. GZMB binding to Zofenopril-calcium formed hydrogen bonds with LYS-113 and ARG-87, while AGI-6780 binding formed no hydrogen bonds. Small molecules in the binding of STAT1 to Calcipotriol formed hydrogen bonds with GLU-353 and GLN-271, and small molecules in the binding of TLR7 to Zofenopril-calcium formed hydrogen bonds mainly with LYS-464. The majority of receptor and ligand binding energies are less than -7 kcal.mol^-1^, indicating that the target protein and active ingredient can bind spontaneously with high affinity and stable conformation, and thus small molecule medicines are likely to act on these targets. To illustrate the molecular interactions, we chose the small molecule medication with the lowest binding energy to dock the target for docking visualization (**Supplementary Figures 5A–H**).

**Table 1:** Results of Cmap analysis.

Cmap name	N	Celline	Enrichment	FDR_Q_nlog10
Gabapentin	2	YAPC	-0.94	15.65
Baricitinib	3	HBL1	-0.92	15.65
DPN	3	A549	-0.91	15.65
AGI-6780	2	PC3	-0.9	15.65
Fusaric-acid	3	SKB	-0.9	15.65
Ru-24969	3	MCF7	-0.89	15.65
Calcipotriol	2	HCC515	-0.89	15.65
Fenoterol	2	YAPC	0.89	15.65
Zofenopril-calcium	2	JURKAT	-0.89	15.65
RS-102895	3	A549	-0.89	15.65

### Expression validation of key molecules

**Figure 10:**
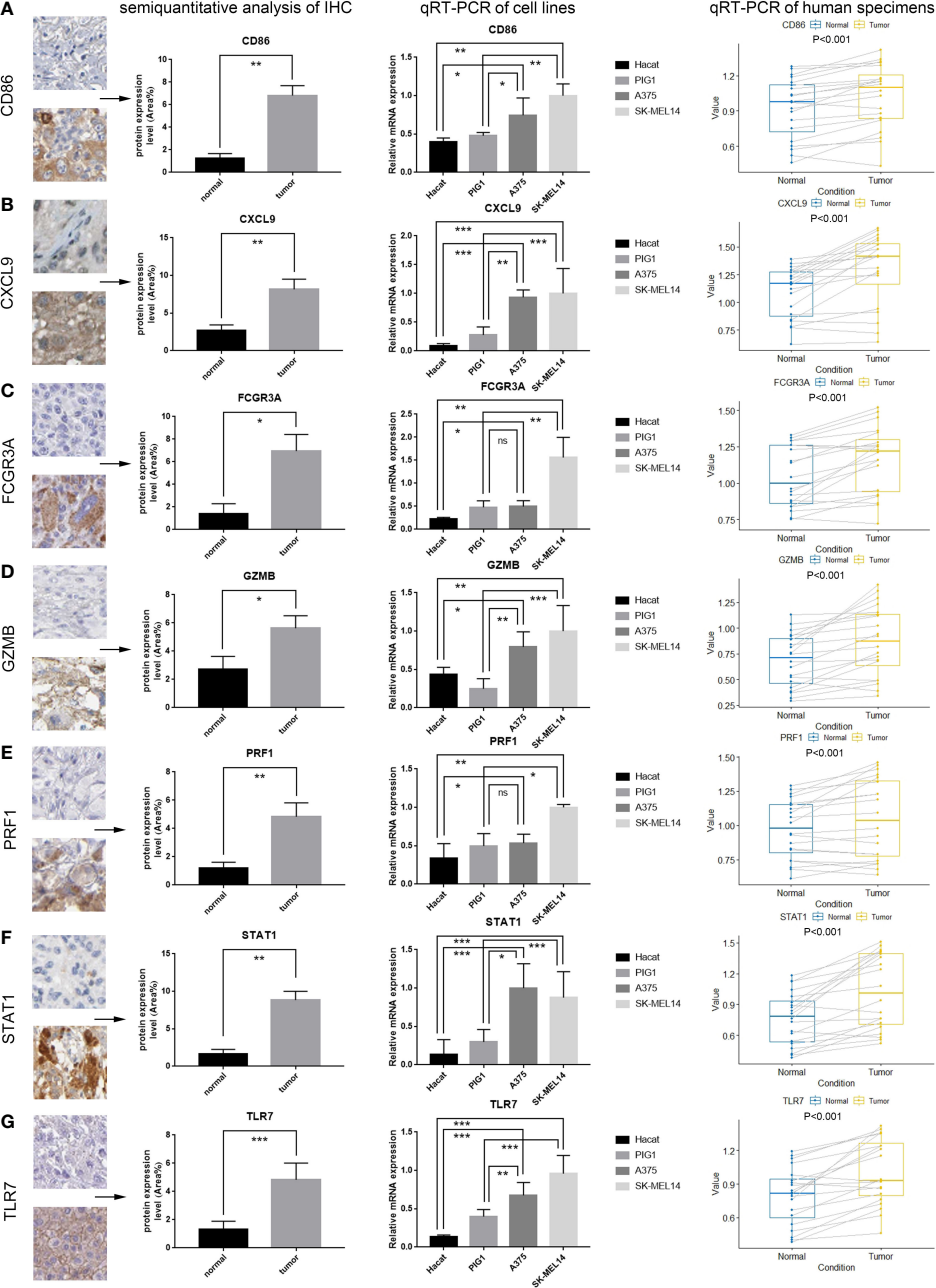
Validation of the mRNA and protein expression of seven key genes. The results of the first column represent the semiquantitative analysis results obtained from the IHC results downloaded from the human protein atlas (see **Figure 3** for a complete view of the immunohistochemistry images). The results of the second column represent the qRT-PCR results from four cell lines (Hacat, PIG1, A375 and SK-MEL 14). The results of the third column represent the qRT-PCR results of tissue specimens from 20 patients, which were taken from normal skin and cutaneous melanoma. **(A–G)** Results of semiquantitative analysis of IHC, qRT-PCR of cell lines, qRT-PCR of human specimens for seven key genes, including CD86, CXCL9, FCGR3A, GZMB, PRF1, STAT1, TLR7 (ns, not significant, *P<0.05, **P <0.01, ***P <0.001).

We utilized semi-quantitative analysis to validate the differential expression of these proteins in normal skin tissues and CM after selecting one appropriate field of view (the first column of **Figure 10**). qRT-PCR was subsequently utilized to confirm the differential expression of these seven essential genes in cell lines and human specimens. The mRNA expression of seven essential genes was significantly higher in the A375 and SK-Mel-14 cell lines than in the Hacat and PIG1 cell lines (the second column of **Figure 10**). Similarly, we found that the mRNA expression of seven key genes in patients was much higher in CM than in normal skin tissue (the third column of **Figure 10**). Using experimental validation at the mRNA and protein levels, we determined that CD86, CXCL9, FCGR3A, GZMB, PRF1, STAT1, and TLR7 were differentially expressed in normal skin tissues and CM and inferred that these key molecules could be potentially critical targets for the treatment of CM.

## Discussion

In recent years, chemotherapy, immunotherapy, and targeted therapies have been utilized to treat patients with advanced CM. Nonetheless, there are still issues such as high drug resistance, low drug sensitivity, and poor prognosis. Recent advancements in sequencing technology have opened up new avenues for methodically deciphering key genes and epigenetic alterations in various types of CM. In this study, we combined immune-related gene sets and other datasets to explore the complex integrative roles of multiple key molecules on TME infiltration and heterogeneity. We not only revealed the potential mechanisms of TME anti-tumor immune response but also screened potential biological therapeutic targets and performed small molecular drug prediction.

Numerous CM prognostic models created based on different aspects have shown distinct therapeutic applicability in recent years. However, few CM models are available for immune genes, immunotherapy, and small molecule drug prediction. Zhang’s study established a five-mRNA prognostic signature to predict CM prognosis and immunotherapy response (33). Liu’s study constructed a ten ferroptosis-related prognostic DEGs signature to predict the prognosis of CM (34). Shi’s study established an epithelial-mesenchymal transition (EMT)-related gene pairs (ERGPs) signature that could be potentially used in a clinical setting as a genetic biomarker for risk stratification of CM patients (35). Liu’s study constructed an Fc Receptor-like (FCRL) prognostic signature, which could act as a biomarker to predict the prognosis of CM patients (36). Yang’s study established a comprehensive glycolysis and immune (CIGI) model, which could be served as an independent prognostic factor for CM patients (37). Wu’s study constructed an m^1^ A-, m^5^ C- and m^6^ A-related signature, which may be a promising biomarker for future CM research (38). All the above models have been constructed from different perspectives of CM and can provide valuable references for clinical decision-making. The AUC values of our model are better than the above models, indicating that our model is better in predictive performance and has some potential for clinical application. We also compared other recent CM prognostic signatures constructed from different perspectives (39–41), and we found that the prognostic signature in this study showed better clinical predictive performance in comparison.

In the present study, we evaluated the key molecules influencing patient prognosis utilizing 469 CM samples and 556 normal skin samples from multiple database gene sets. We observed significant differences in immune-related pathways by examining the genetic changes between normal skin tissues and CM tissues. We employed seven key molecules with high interaction as the foundation of the prediction model. The expression of these seven essential genes was dramatically elevated in tumor tissue as protective genes and was highly correlated with a significantly more extended survival period. Through immune-related analysis, we determined that CM had much higher immune cell infiltration levels than normal skin and significantly higher total immunological and mesenchymal activity, which altered the TME’s infiltration pattern. We used the LASSO COX regression model to develop the riskScore signature. We discovered that low-risk patients had more significant TME immune cell infiltration and a longer survival time. We also discovered that riskScore could be utilized as an independent biomarker to assess patient prognosis by multivariate COX regression. We integrated riskScore and independent clinical prognostic markers to generate nomogram plots that displayed excellent predictive performance. In the IMvigor210 group receiving anti-PD-L1 therapy, we observed a significant therapeutic benefit with increased survival time and enhanced clinical response in low-risk patients compared to those at high risk, which demonstrates the good predictive performance of our riskScore signature. Moreover, using small molecule drug screening and molecular docking, Gabapentin and Baricitinib were discovered as prospective small-molecule medicines to treat CM. Finally, IHC and qRT-PCR were performed to confirm the expression of important molecules.

CM is considered one of the most immunogenic tumors due to its high mutational load, and many immune cells infiltrate. Immune cell infiltration is an essential protective mechanism of the organism and forms the basis for overt cellular therapies and cellular vaccines to treat cancer. All seven genes in our riskScore signature are mutated in CM patients, with CD86 having the highest frequency of mutations. Studies have shown that CD86 gene polymorphisms in miRNA are associated with the risk of malignancies such as pancreatic, cervical, and colon cancers. Therefore, we hypothesize that CD86 might be one of the potential targets for CM therapy (42). The riskScore signature consists of seven key molecules, CD86, CXCL9, FCGR3A, GZMB, PRF1, STAT1, and TLR7, all of which are expressed up-regulated in CM as protective molecules. CD86 (cluster of differentiation 86), a member of the immunoglobulin superfamily, interacts with the inducer CD28 and the inhibitor CTLA4 and functions as a crucial cofactor in the stimulation of T-lymphocyte proliferation and IL-2 production (43). CTLA4, an immunological checkpoint molecule, can influence the TME of CM by binding to B7 (CD80/CD86) molecules on melanoma antigen-presenting cells to down-regulate T cell activation (44). The chemokine CXCL9 (C-X-C motif chemokine ligand 9) correlates positively with CD8^+^T cell infiltration in solid malignancies (45, 46). CXCL9 is abundantly expressed in several solid tumors, including CM, and it stimulates the infiltration of CD4^+^T and CD8^+^T lymphocytes into tumor cell regions, hence boosting the response of cytotoxic T lymphocytes and destroying tumor cells (47, 48). FCGR3A (Fc fragment of IgG receptor IIIa) encodes the receptor for the Fc region of immunoglobulin G. FCGR3A interacts with FCGR1A in numerous pathophysiological processes and is substantially related with overall survival (OS) in CM, renal clear cell carcinoma, and other malignancies (49). Granzyme B (GZMB) is an exogenous serine protease generated from granules released by cytotoxic lymphocytes (CTLs) and natural killer cells (NK) (50). GZMB has been discovered to be related with NK cell treatment in individuals with CM. By evaluating NK cells in the blood of CM patients, it was discovered that NK cells entering metastatic melanoma tissue have a diminished cytotoxic capacity due to decreased expression of GZMB and perforin (51). PRF1 (perforin 1) encodes a protein structurally similar to complement C9, which plays a crucial function in immunity (52). According to research, tumors from CM patients treated with the anti-PD1 medication nivolumab had significantly greater levels of PRF1, CD8, and GZMA, as well as an improved TBX21/GATA3 ratio. This suggests that PRF1 mediates tumor-infiltrating T lymphocytes (TIL) oligoclonal amplification-enhanced Th1 (helper T cell type I)-skewed cellular immunity during nivolumab treatment (53). STAT1 (signal transducers and activators of transcription 1) is a family of cytosolic proteins that, upon activation, can translocate to the nucleus and bind DNA, which has dual signal transduction and transcriptional control functions (54, 55). Hypermethylation in the promoter region of the SOCS3 gene was discovered to reduce SOCS3 protein expression in some CM patients. The greater the sensitivity of melanoma cells to IFN-γ, the lower the expression of SOCS3, and the lowering of SOCS3 expression in melanoma cells by IFN-γ may significantly stimulate the production of STAT1 (56). TLR7 (toll-like receptors 7) is an endosomal pattern recognition receptor, when activated, causes type I interferons and inflammatory reactions (57). TLR7 plays a crucial role in activating both natural and acquired immune responses and has an activating effect on virtually all cells engaged in the tumor immune response (58). Chemically coupling ibrutinib with TLR7 receptor agonists to produce novel immune-targeting complexes termed GY161 increased CD8^+^T cell levels in spleen and tumor *in vivo*. GY161 inhibited the growth of B16 melanoma cell-derived tumors and prolonged the survival time of mice (59).

The significance of the TME in tumor development is critical. Several studies have shown that various immune cells can serve as tumor promoters or tumor antagonists in various malignancies (60, 61). Therefore, further investigation on immune infiltration in TME is required to better understand the link between immunological components and tumor progression. After constructing a riskScore model to divide the high- and low-risk groups, we discovered that the expression of seven protective key molecules was significantly lower in the high-risk group. In contrast, immune cell infiltration was significantly lower in the high-risk group compared to the low-risk group. We found that the low-risk group had higher levels of CD4^+^ and CD8^+^ T cells. CD8^+^ T cells eliminate tumor cells based on cell differentiation and invasion. They can differentiate into effector and cytotoxic T cells to perform anti-tumor actions in the tumor-infiltrating microenvironment (62). In secondary lymphoid organs, CD4^+^ T cells can inhibit or stimulate the activity of anti-tumor cytotoxic T cells, hence modulating tumor cells. Tumor infiltration lymphocytes (TILs) in CM are a potential immunotherapy target in the future (63). Inflammation also plays a vital role in TME and tumor formation. Since Virchow proposed in 1863 that tumor formation originates from chronic inflammation, numerous studies have confirmed that some tumors are closely associated with chronic inflammation. We discovered significantly higher inflammation-associated immune cell infiltration in CM than in normal skin, indicating that inflammation in the TME has a pro-tumor effect. It can assist the proliferation and survival of cancer cells as well as promote angiogenesis and metastasis (64). Our riskScore signature demonstrated significantly higher inflammation-associated immune cell infiltration in the low-risk group compared to the high-risk group. The low-risk group had higher MDSCs, TILs (CD4^+^ T cells and CD8^+^ T cells), TAMs, dendritic cells, neutrophils, and mast cells. MDSCs are immature bone marrow cells that suppress natural and adaptive immunity and evade immune surveillance (65). TAMs can promote tumor development while also increases vascular growth and invasive metastasis (66). Neutrophils and TILs can play a role in killing tumor cells. B lymphocytes and mast cells also play an essential role in immune-mediated tumor growth. Additionally, macrophages and dendritic cells play antigen presentation and T-cell activation roles, as well as immunosuppressive functions in tumors (67).

The innovative usage of traditional drugs has now become an important strategy for antineoplastic drug development. The discovery of potential mechanisms of conventional drugs can save time and money while improving drug administration security. According to the CMAP database, gabapentin and baricitinib are promising treatments for CM. Gabapentin, whose mechanism of action is currently unknown, is commonly believed to modify the GABA metabolic pathway in patients with circumscribed seizures that are not adequately controlled or tolerated by traditional antiepileptic medicines. Several investigations have verified the anticancer effects of Gabapentin in recent years. Gabapentin may achieve anti-melanoma effects in mice by reducing cell proliferation, CCL2 production, and calcium influx (68). In recent years, it has been demonstrated that thiamine-dependent enzymes (TDEs) are frequently tumor-related targets due to their control of metabolic pathways that are frequently altered in cancer. Gabapentin can impede the growth of TDEs, resulting in a tumor-killing mechanism of toxicity (69). We found that Gabapentin most likely acts through STAT1 and TLR7 to achieve anti-tumor effects by altering the immune infiltration content of TILs such as CD8^+^ T cells in TME to inhibit the proliferation and invasion of CM cells using molecular docking analysis and a summary of key molecules mentioned above.

There are some limitations to this study. First, the clinical parameters integrated with this study may not be comprehensive due to the limited clinical information in the public dataset, leading to potential bias in our construction of the riskScore signature. Second, the plasticity of immune cells or other disease-induced cellular changes may bias the analysis results. Moreover, we constructed the riskScore signature mainly based on the TCGA database. Given the differences in database compatibility, we should take caution while employing this study’s riskScore signature for testing in other databases.

In conclusion, the riskScore signature developed in this study can be utilized as an independent and reliable biomarker to predict the prognosis of individuals with CM. In addition, we screened and predicted small-molecule pharmaceuticals. This study not only provides the riskScore signature that can predict patient prognosis and assess the heterogeneity and complexity of TME cell infiltration but also contributes to the development and guidance of novel immune combination therapy strategies and the promotion of the development of personalized tumor immunotherapy and precision medicine.

## Data availability statement

The original contributions presented in the study are included in the article/**supplementary material**. Further inquiries can be directed to the corresponding authors.

## Ethics statement

The studies involving human participants were reviewed and approved by Chinese PLA General Hospital. The patients/participants provided their written informed consent to participate in this study.

## Author contributions

JX performed majority contributions to research design, data analysis and article writing. JX and ZJ conceived and designed the study, YL and YH suggested ideas and steps for the article, and participated in the revision of article. All authors contributed to the article and approved the submitted version.

## Conflict of interest

The authors declare that the research was conducted in the absence of any commercial or financial relationships that could be construed as a potential conflict of interest.

## Publisher’s note

All claims expressed in this article are solely those of the authors and do not necessarily represent those of their affiliated organizations, or those of the publisher, the editors and the reviewers. Any product that may be evaluated in this article, or claim that may be made by its manufacturer, is not guaranteed or endorsed by the publisher.

## Supplementary material

Supplementary Figure 1 the flowchart of this study.

Supplementary Figure 2
**(A)** Survival analyses for high and low risk score groups in anti-PD-L1 immunotherapy cohort using Kaplan-Meier curves. **(B)** The difference of riskScore in different anti-PD-L1 clinical response groups. CR, complete response. PD, progressive disease. PR, partial response. SD, stable disease. **(C)** The proportion of patients with response to PD-L1 blockade therapy in high or low riskScore groups.

Supplementary Figure 3 The riskScore signature is associated with chemotherapy and targeted therapy sensitivity. **(A)** Axitinib. **(B)** Bexarotene. **(C)** Bicalutamide. **(D)** Bleomycin. **(E)** Camptothecin. **(F)** Cytarabine. **(G)** Dasatinib. **(H)** Doxorubicin. (I) Elesclomol. **(J)** Erlotinib. **(K)** Gefitinib. **(L)** Imatinib. **(M)** Lapatinib. **(N)** Metformin. **(O)** Midostaurin. **(P)** Nilotinib. **(Q)** Pazopanib. **(R)** Sorafenib. **(S)** Sunitinib. **(T)** Tipifarnib.

Supplementary Figure 4 Chemical structure depiction of the top ten most significant drugs. **(A)** AGI-6780. **(B)** Baricitinib. **(C)** Calcipotriol. **(D)** DPN. **(E)** Fenoterol. **(F)** Fusaric acid. **(G)** Gabapentin. **(H)** RS-102895. **(I)** Zofenopril calcium. **(J)** Ru-24969.

Supplementary Figure 5 Docking diagram of small molecular drugs with targets. (A) CD86-Baricitinib. **(B)** FCGR3A-Zofenopril calcium. **(C)** GZMB-Zofenopril calcium. **(D)** GZMB-AGI 6780. **(E)** STAT1-Gabaoentin. **(F)** TLR7-Gabaoentin. **(G)** STAT1-Calcipotriol. (H) TLR7-Zofenopril calcium.
