# Supplementary figures and images for: Construction of immunotherapy-related prognostic gene signature and small molecule drug prediction for cutaneous melanoma
Source: Front Oncol. 2022 Jul 25;12:939385. doi: 10.3389/fonc.2022.939385 (PMC9358033; doi:10.3389/fonc.2022.939385)

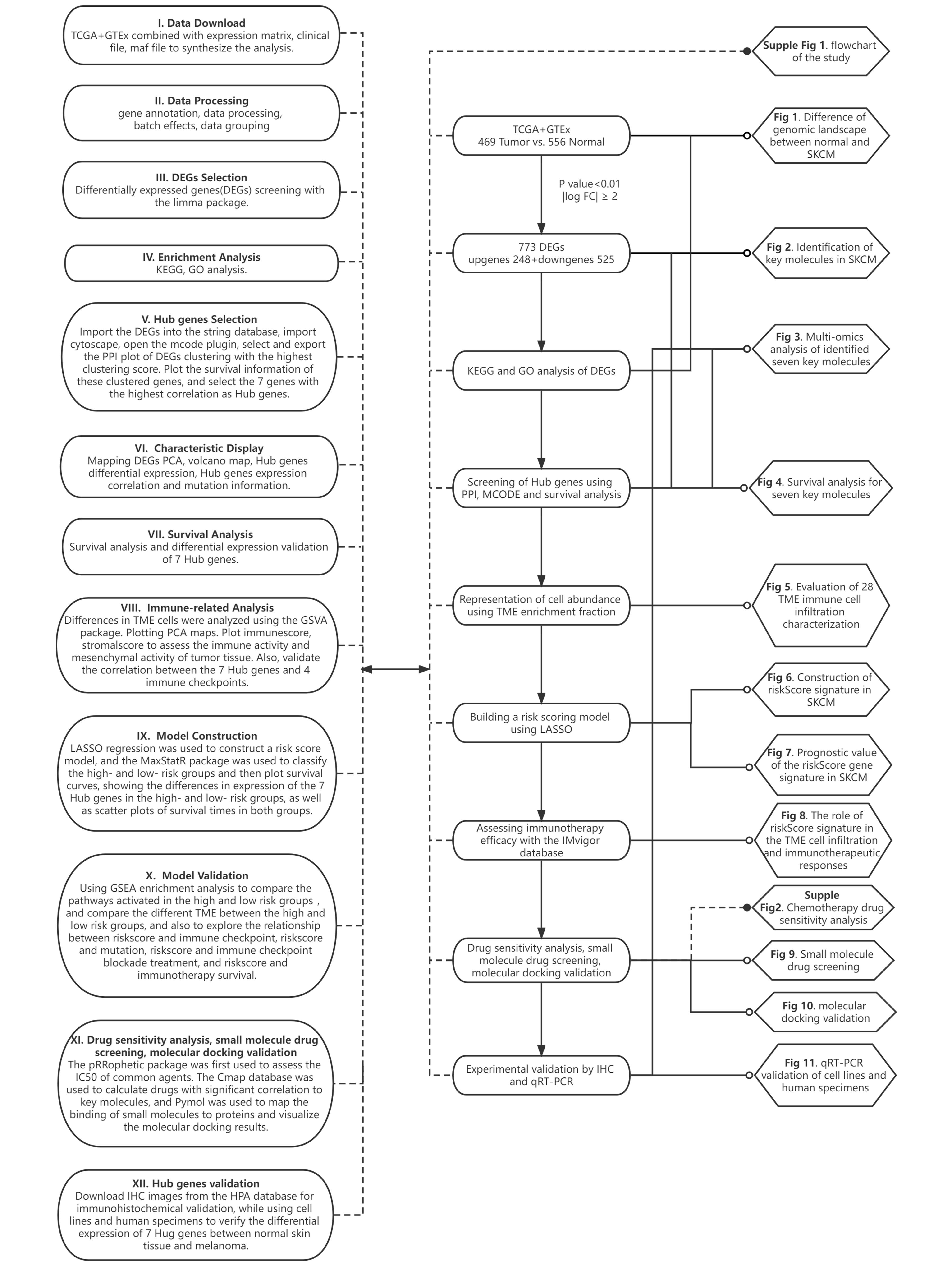

Supplement: Supplementary Figure 1 — the flowchart of this study. [file Image_1.jpeg]

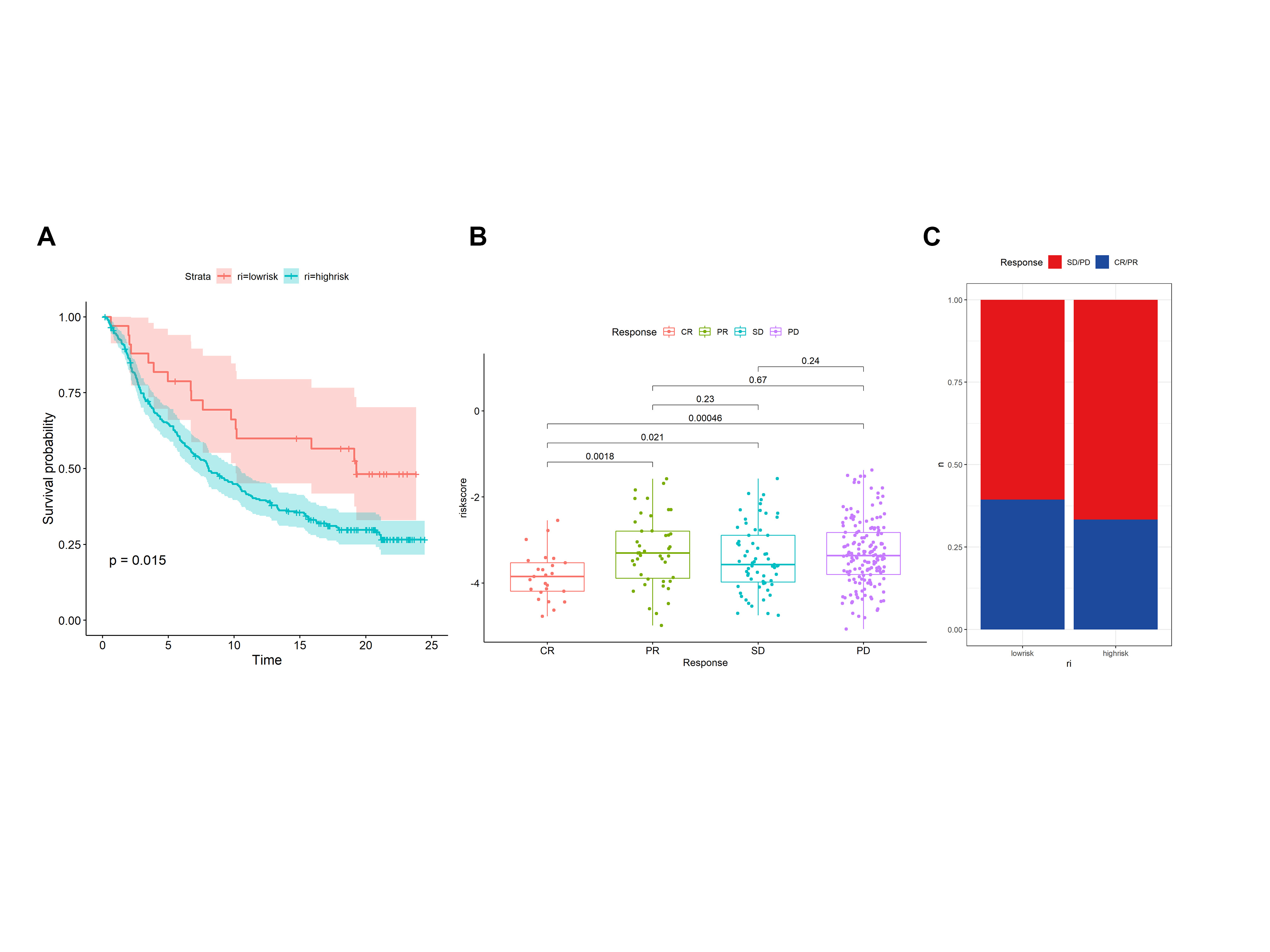

Supplement: Supplementary Figure 2 — (A) Survival analyses for high and low risk score groups in anti-PD-L1 immunotherapy cohort using Kaplan-Meier curves. (B) The difference of riskScore in different anti-PD-L1 clinical response groups. CR, complete response. PD, progressive disease. PR, partial response. SD, stable disease. (C) The proportion of patients with response to PD-L1 blockade therapy in high or low riskScore groups. [file Image_2.jpeg]

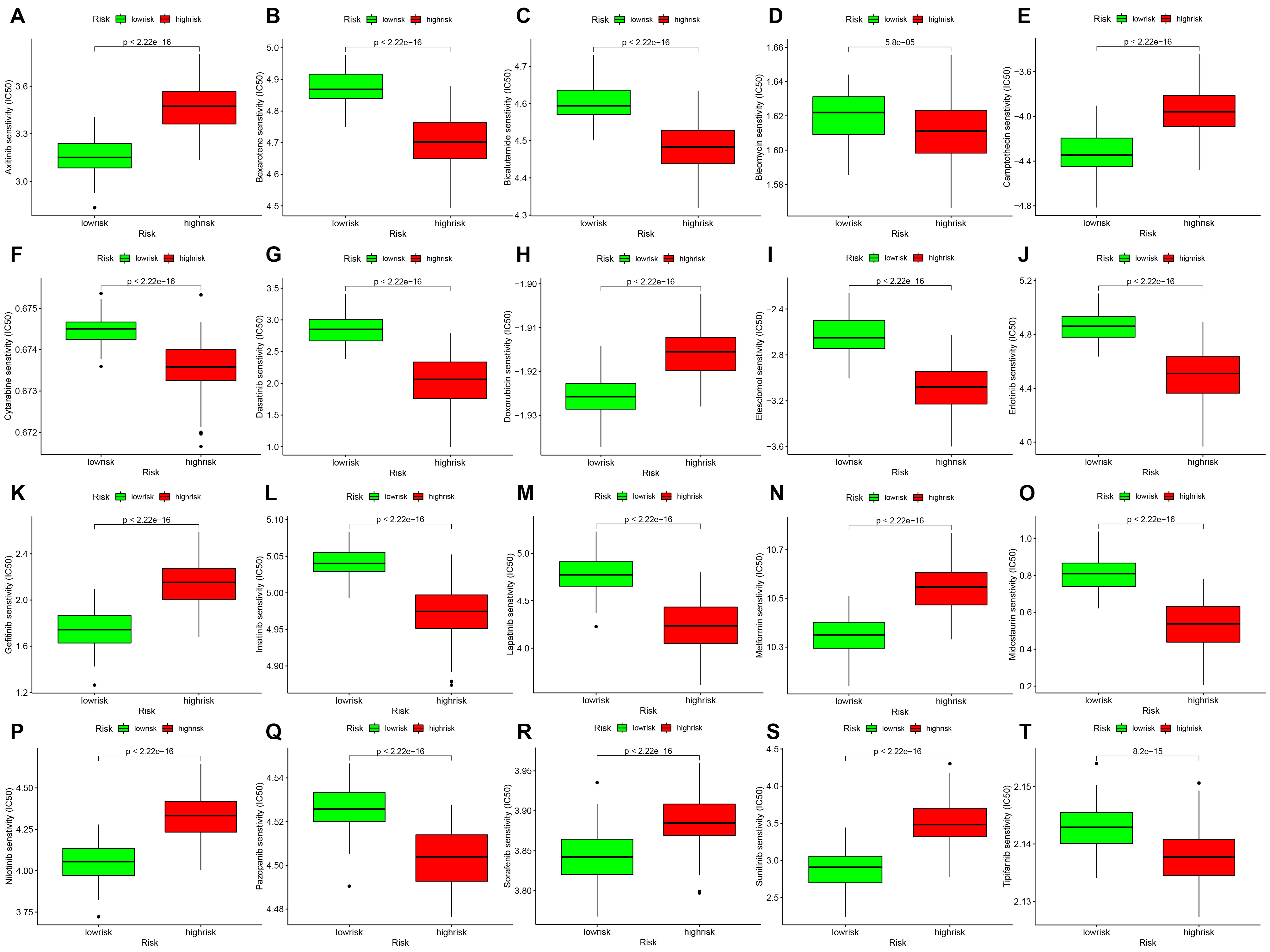

Supplement: Supplementary Figure 3 — The riskScore signature is associated with chemotherapy and targeted therapy sensitivity. (A) Axitinib. (B) Bexarotene. (C) Bicalutamide. (D) Bleomycin. (E) Camptothecin. (F) Cytarabine. (G) Dasatinib. (H) Doxorubicin. (I) Elesclomol. (J) Erlotinib. (K) Gefitinib. (L) Imatinib. (M) Lapatinib. (N) Metformin. (O) Midostaurin. (P) Nilotinib. (Q) Pazopanib. (R) Sorafenib. (S) Sunitinib. (T) Tipifarnib. [file Image_3.jpeg]

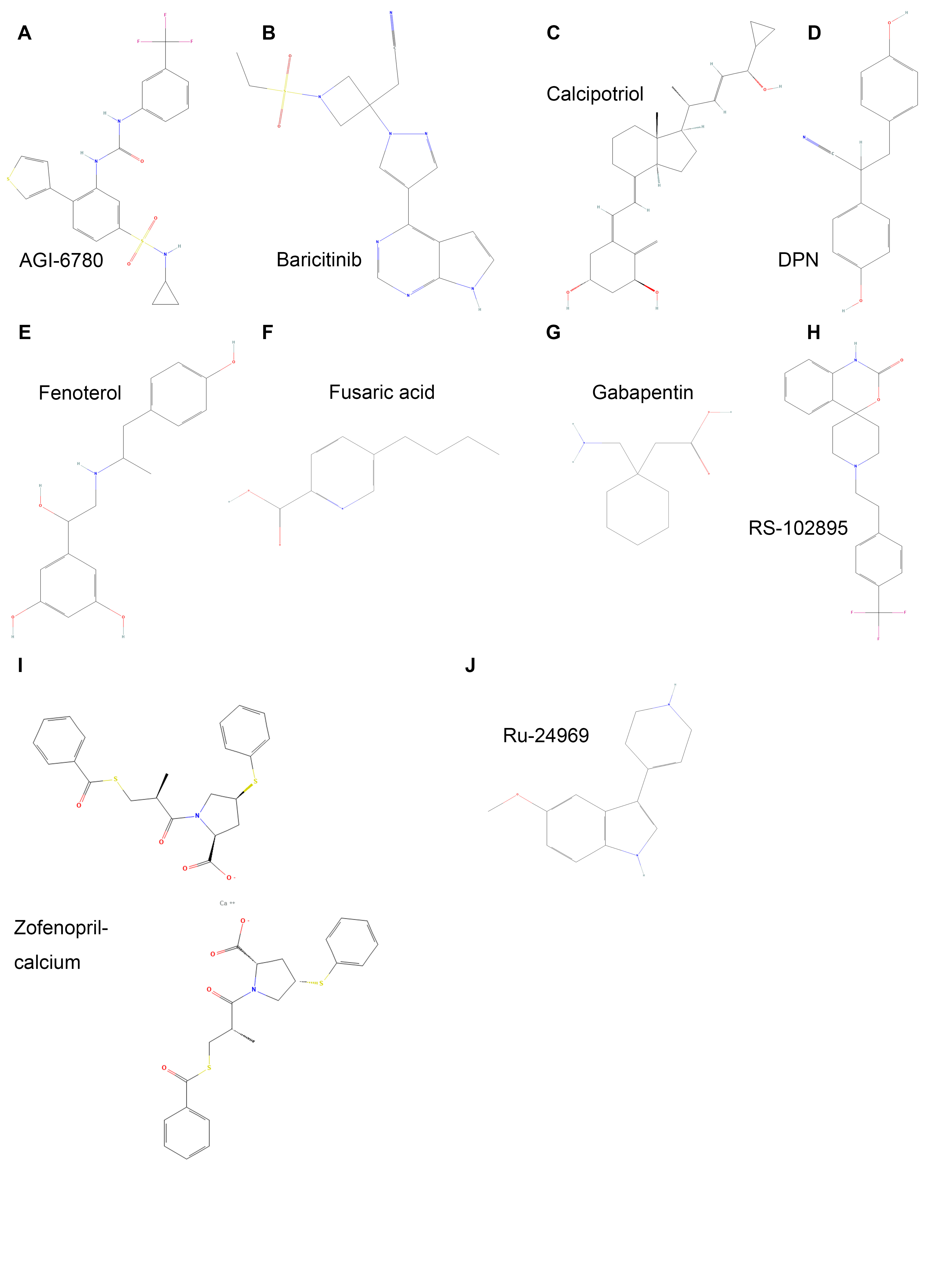

Supplement: Supplementary Figure 4 — Chemical structure depiction of the top ten most significant drugs. (A) AGI-6780. (B) Baricitinib. (C) Calcipotriol. (D) DPN. (E) Fenoterol. (F) Fusaric acid. (G) Gabapentin. (H) RS-102895. (I) Zofenopril calcium. (J) Ru-24969. [file Image_4.jpeg]

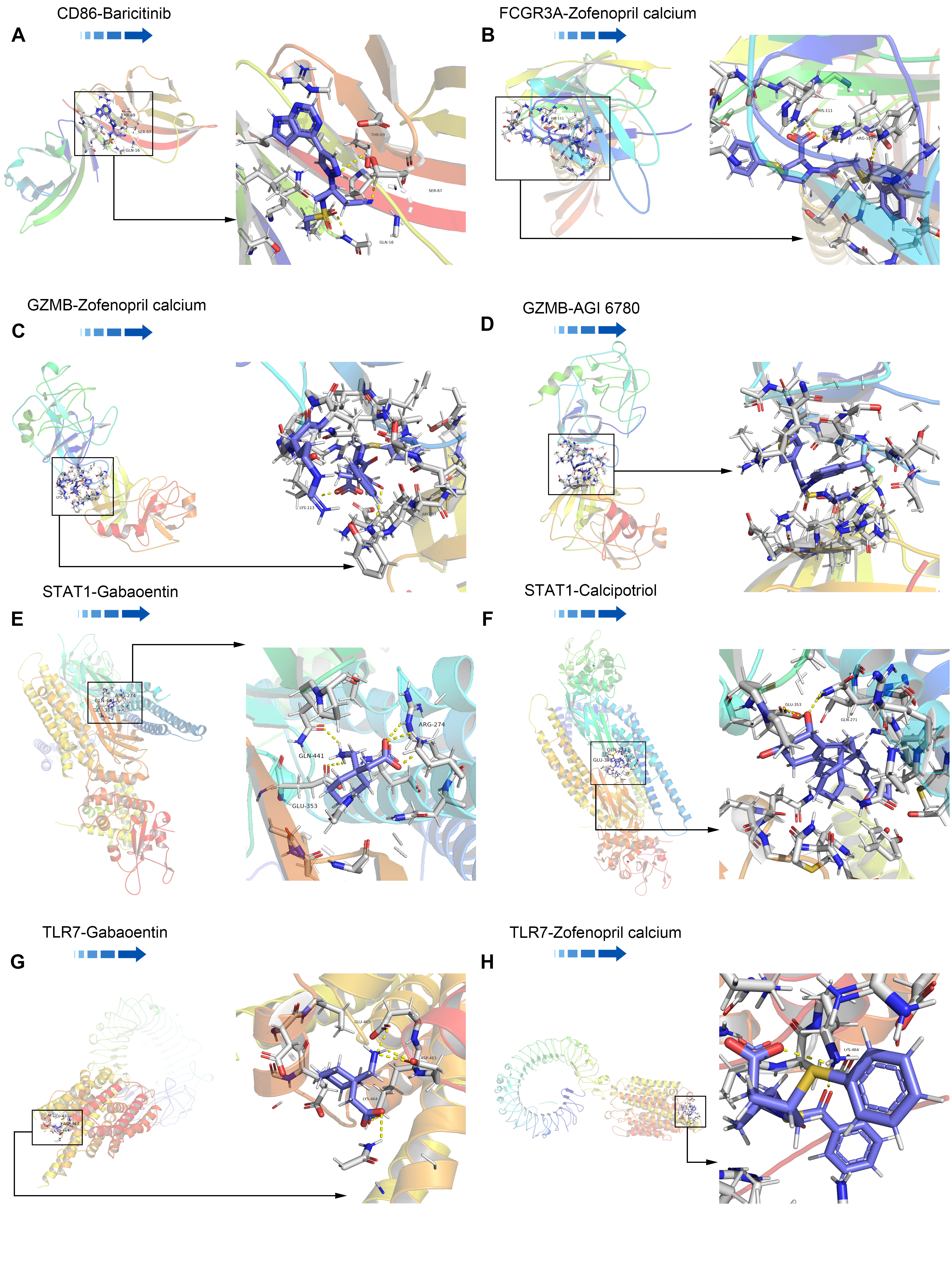

Supplement: Supplementary Figure 5 — Docking diagram of small molecular drugs with targets. (A) CD86-Baricitinib. (B) FCGR3A-Zofenopril calcium. (C) GZMB-Zofenopril calcium. (D) GZMB-AGI 6780. (E) STAT1-Gabaoentin. (F) TLR7-Gabaoentin. (G) STAT1-Calcipotriol. (H) TLR7-Zofenopril calcium. [file Image_5.jpeg]
