# Supplementary material for: Construction of immunotherapy-related prognostic gene signature and small molecule drug prediction for cutaneous melanoma
Source: Front Oncol. 2022 Jul 25;12:939385. doi: 10.3389/fonc.2022.939385 (PMC9358033; doi:10.3389/fonc.2022.939385)
Supplement: Supplementary file 6 [file Table_1.docx]

**Supplementary Table 1**: The base sequence of primers

| **Primer name** | **Base sequence (5’ to 3’)** |
| --- | --- |
| STAT1-F | CAGCTTGACTCAAAATTCCTGGA |
| STAT1-R | TGAAGATTACGCTTGCTTTTCCT |
| CXCL9-F | CCAGTAGTGAGAAAGGGTCGC |
| CXCL9-R | AGGGCTTGGGGCAAATTGTT |
| CD86-F | CTGCTCATCTATACACGGTTACC |
| CD86-R | GGAAACGTCGTACAGTTCTGTG |
| FCGR3A-F | CCTCCTGTCTAGTCGGTTTGG |
| FCGR3A-R | TCGAGCACCCTGTACCATTGA |
| TLR7-F | TCGTGGACTGCACAGACAAG |
| TLR7-R | GGTATGTGGTTAATGGTGAGGGT |
| PRF1-F | GTGGGACAATAACAACCCCAT |
| PRF1-R | TGGCATGATAGCGGAATTTTAGG |
| GZMB-F | TACCATTGAGTTGTGCGTGGG |
| GZMB-R | GCCATTGTTTCGTCCATAGGAGA |

**Supplementary Table 2**: The basic information of patient specimens

| **Number** | **Age** | | **Gender** | **Site of lesion occurrence** | **TNM staging**  **(ACJJ Staging Eighth Edition)** |
| --- | --- | --- | --- | --- | --- |
| 1 | 51 | male | | Face | T2bN0M0-IIA |
| 2 | 46 | male | | Neck | T1aN1bM0-IIIB |
| 3 | 49 | male | | Anterior chest | T3aN2bM0-IIIB |
| 4 | 42 | male | | Face | T2aN0M0-IB |
| 5 | 57 | male | | Face | T2aN1aM0-IIIA |
| 6 | 52 | male | | Upper Extremity | T2bN1aM0-IIIB |
| 7 | 42 | male | | Palm | T2bN0M0-IIA |
| 8 | 63 | male | | Neck | T3bN3bM0-IIIC |
| 9 | 54 | male | | Face | T2bN1bM0-IIIB |
| 10 | 50 | male | | Upper Extremity | T3aN3aM0-IIIC |
| 11 | 46 | male | | Upper Extremity | T2bN0M0-IIA |
| 12 | 49 | male | | Face | T2bN1aM0-IIIB |
| 13 | 54 | male | | Neck | T2bN1aM0-IIIB |
| 14 | 57 | male | | Anterior chest | T3bN3bM0-IIIC |
| 15 | 52 | male | | Palm | T2bN1bM0-IIIB |
| 16 | 54 | female | | Face | T2bN0M0-IIA |
| 17 | 46 | female | | Palm | T1aN0M0-IA |
| 18 | 51 | female | | Face | T2aN1bM0-IIIB |
| 19 | 50 | female | | Anterior chest | T2bN0M0-IIA |
| 20 | 48 | female | | Neck | T1bN0M0-IA |
